# Supplementary material for: Sustainable One-Step Solid-State Synthesis of Antibacterially Active Silver Nanoparticles Using Mechanochemistry
Source: Nanomaterials (Basel). 2020 Oct 25;10(11):2119. doi: 10.3390/nano10112119 (PMC7692266; doi:10.3390/nano10112119)
Supplement: Supplementary file 1 [file nanomaterials-10-02119-s001.pdf]

Electronic Supplementary Information for the paper

Sustainable one-step-solid state synthesis of antibacterially active silver nanoparticles using mechanochemistry

by

M. Kováčová [a]*, N. Daneu [b], Ľ. Tkáčiková [c], E. Dutková [a], R. Búreš [d], M. Stáhorský [a], Z. Lukáčová Bujňáková [a], , and M. Baláž[a]*

[a]*Department of Mechanochemistry, Institute of Geotechnics, Slovak Academy of Sciences, Watsonova 45, 04001 Košice, Slovakia*

[b]*Advanced Materials Department, Jozef Štefan Institute, Jamova 39, 1000 Ljubljana, Slovenia*

[c]*Department of Microbiology and Immunology, University of Veterinary Medicine and Pharmacy, Komenského 73, 04181 Košice, Slovakia*

[d] *Institute of Materials Research, Slovak Academy of Sciences, Košice, Slovakia;*

*Corresponding authors e-mails: kovacovam@saske.sk, balazm@saske.sk

Milling of pure $AgNO_3$

The XRD pattern of pure $AgNO_3$ subjected to milling under the same conditions as the Ag:plant mixtures showing just reflections of $AgNO_3$ (no reduction to Ag^0) can be seen in Fig. S1.

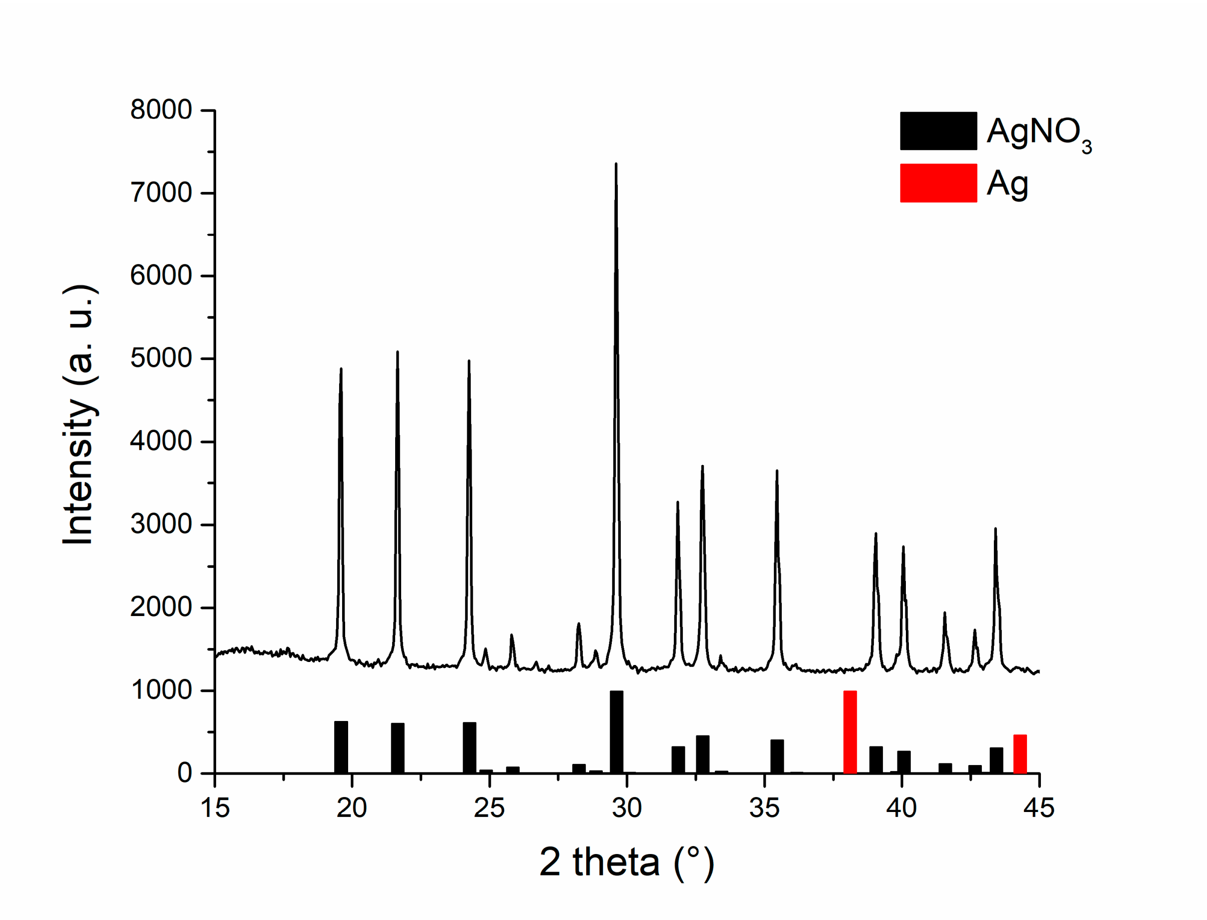

Figure S1: XRD pattern of milled $AgNO_3$

Selected area diffraction patterns obtained during the TEM analysis

As outlined in the main body of the paper, SAED was used to confirm the presence of Ag^0 in all samples shown in Figure 5. The SAED patterns are shown in Fig. S2.

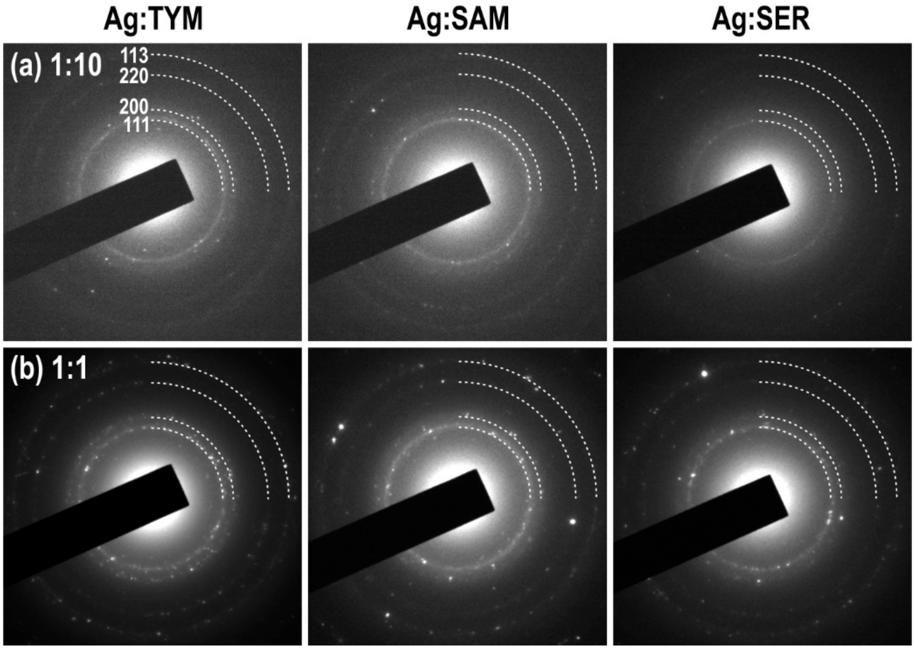

Figure S2: SAED patterns of the samples with Ag:plant ratio (a) 1:10 and (b) 1:1. Dotted ring patterns indicate random orientation of Ag NPs. Only reflections from face-centered cubic Ag are present (indices marked in the Ag:TYM 1:10 pattern) The patterns in the 1:10 samples are composed of finer spots indicating smaller average particle size, whereas in the 1:1 samples brighter reflections from few larger Ag NPs are observed.
